# CXCL13/CXCR5 axis facilitates endothelial progenitor cell homing and angiogenesis during rheumatoid arthritis progression

**DOI:** 10.1038/s41419-021-04136-2

**Published:** 2021-09-13

**Authors:** Chun-Hao Tsai, Chao-Ju Chen, Chi-Li Gong, Shan-Chi Liu, Po-Chun Chen, Chien-Chung Huang, Sung-Lin Hu, Shih-Wei Wang, Chih-Hsin Tang

**Affiliations:** 1 Department of Sports Medicine, College of Health Care, China Medical University, Taichung, Taiwan; 2 Department of Orthopedic Surgery, China Medical University Hospital, Taichung, Taiwan; 3 School of Medicine, China Medical University, Taichung, Taiwan; 4 Department of Medical Education and Research, China Medical University Beigang Hospital, Yunlin, Taiwan; 5 Translational Medicine Center, Shin-Kong Wu Ho-Su Memorial Hospital, Taipei, Taiwan; 6 Division of Immunology and Rheumatology, Department of Internal Medicine, China Medical University Hospital, Taichung, Taiwan; 7 Department of Family Medicine, China Medical University Hsinchu Hospital, Hsinchu, Taiwan; 8 Department of Medicine, MacKay Medical College, New Taipei City, Taiwan; 9 Graduate Institute of Natural Products, College of Pharmacy, Kaohsiung Medical University, Kaohsiung, Taiwan; 10 Institute of Biomedical Sciences, Mackay Medical College, Taipei, Taiwan; 11 Chinese Medicine Research Center, China Medical University, Taichung, Taiwan; 12 Department of Biotechnology, College of Health Science, Asia University, Taichung, Taiwan

**Keywords:** miRNAs, Rheumatoid arthritis

## Abstract

Angiogenesis is a critical process in the formation of new capillaries and a key participant in rheumatoid arthritis (RA) pathogenesis. The chemokine (C-X-C motif) ligand 13 (CXCL13) plays important roles in several cellular functions such as infiltration, migration, and motility. We report significantly higher levels of CXCL13 expression in collagen-induced arthritis (CIA) mice compared with controls and also in synovial fluid from RA patients compared with human osteoarthritis (OA) samples. RA synovial fluid increased endothelial progenitor cell (EPC) homing and angiogenesis, which was blocked by the CXCL13 antibody. By interacting with the CXCR5 receptor, CXCL13 facilitated vascular endothelial growth factor (VEGF) expression and angiogenesis in EPC through the PLC, MEK, and AP-1 signaling pathways. Importantly, infection with CXCL13 short hairpin RNA (shRNA) mitigated EPC homing and angiogenesis, articular swelling, and cartilage erosion in ankle joints of mice with CIA. CXCL13 is therefore a novel therapeutic target for RA.

## Introduction

Rheumatoid arthritis (RA) is one of the most common autoimmune disorders, characterized by the accumulation of inflammatory cytokines in the synovial joint, resulting in pannus formation, cartilage degradation, and bone destruction [1]. Angiogenesis is a critical driver of RA progression, whereby pre-existing vessels promote the entry of blood-derived leukocytes into the synovial tissues to facilitate and potentiate inflammation [2].

Endothelial progenitor cells (EPCs) develop from bone marrow-derived endothelial stem cells, which contain the cell surface markers CD133, CD34, and vascular endothelial growth factor receptor 2 (VEGFR2) and are capable of stimulating postnatal vasculogenesis [3] and angiogenic function [4]. VEGF induces EPC proliferation and migration, and facilitates angiogenesis [4], enabling the development of RA [5, 6]. EPC-dependent angiogenesis, therefore, seems to be a worthwhile treatment target in RA.

The chemokine (C-X-C motif) ligand 13 (CXCL13), also called the B-lymphocyte chemoattractant, plays an important role in multiple cellular functions, such as migration, invasion, motility, proliferation, and apoptosis [7, 8]. CXCL13 is a critical mediator of the homing and activation of cells at lymphoid sites [9]. Overexpression of CXCL13 in lymphoid sites facilitates B-cell infiltration and invasion, leading to increased lymphoid neogenesis [10]. Recent reports describe how CXCL13 regulates different pathogenic processes including inflammatory responses, cancer progression, metastasis, and drug resistance [8, 11]. High levels of CXCL13 expression in serum from patients with early RA compared with serum from healthy controls serve as an early biomarker of disease severity [12]. The proinflammatory cytokines tumor necrosis factor-alpha (TNF-α) and interleukin (IL)-6 increase CXCL13 production, leading to maturation of B-cell follicles within the synovium during RA progression [9, 13]. Moreover, the interaction of CXCL13 with its specific receptor CXCR5 enhances B-cell maturation and the synthesis of antibodies in autoimmune diseases [14].

Angiogenesis is an early and important process in RA. Inhibiting the CXCL13/CXCR5-mediated signaling pathway is a new direction for the treatment of autoimmune disorders [15]. However, how CXCL13 impacts the angiogenic processes associated with RA remains unclear. In this study, we examined whether high levels of CXCL13 expression in patients with RA promote the homing and angiogenesis of human circulating EPCs during RA development and we investigated the signaling pathways that mediate this process.

## Results

### High levels of CXCL13 expression in RA patients induce EPC homing and angiogenesis

**Fig. 1:**
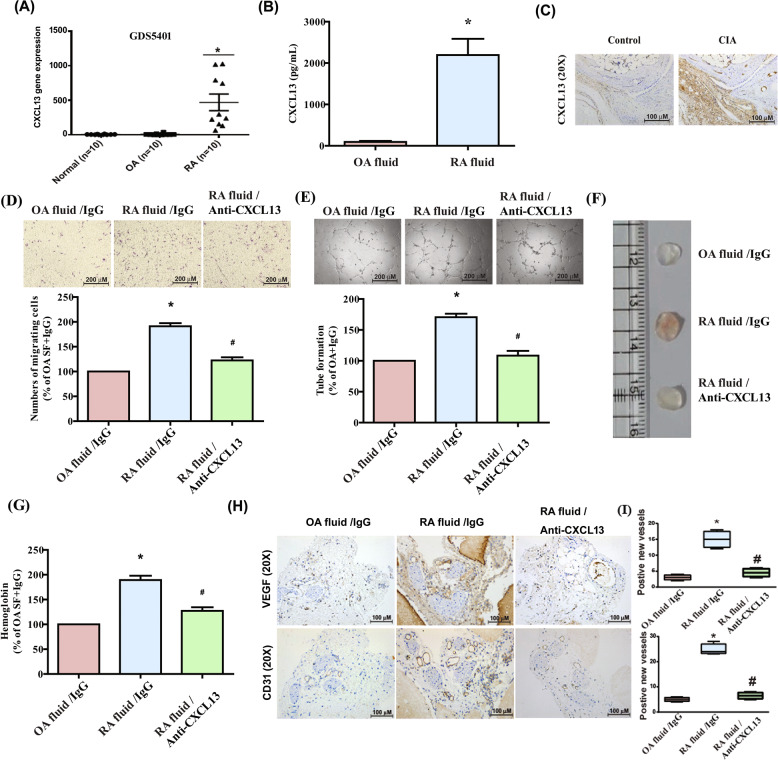
Upregulation of CXCL13 expression in RA promotes EPC homing and angiogenesis. **A** Expression levels of CXCL13 in normal healthy individuals, as well as OA (*n* = 10) and RA (*n* = 10) patients, were retrieved from the GEO database (accession code: GDS5401). **B** CXCL13 levels in OA and RA synovial fluid samples were quantified by the ELISA assay (*n* = 6). **C** IHC staining of CXCL13 in ankle joints of controls and CIA mice (*n* = 6). **D** and **E** RA synovial fluid was treated with or without CXCL13 antibody, then applied to EPCs, before measuring EPC migration (*n* = 6) and tube formation (*n* = 6). **F** Matrigel plugs containing OA synovial fluid plus IgG, RA synovial fluid plus IgG, or RA synovial fluid treated with CXCL13 antibody were subcutaneously injected into the flanks of nude male mice (*n* = 6). **G** After 7 days, the plugs were photographed and hemoglobin levels were quantified. **H** Plug specimens were immunostained with antibodies against CD31 and VEGF (*n* = 6). **I** The new positive new vessels of (**H**) was quantified. *p < 0.05 versus OA synovial fluid; ^#^*p* < 0.05 versus RA synovial fluid.

CXCL13 is associated with the progression of autoimmune diseases, including RA [16]. We, therefore, investigated CXCL13 levels in RA patients. Our analysis of records from the Gene Expression Omnibus (GEO) database revealed higher levels of CXCL13 expression in RA synovial tissue (*n* = 10) compared with those in healthy individuals (*n* = 10) or osteoarthritis (OA) patients (*n* = 10) (Fig. 1A). We also found markedly higher levels of CXCL13 expression in synovial fluid from RA patients compared with OA synovial fluid samples (Fig. 1B), as well as higher levels of CXCL13 expression in collagen-induced arthritis (CIA) mice than in control mice (Fig. 1C). Next, we examined whether the accumulation of CXCL13 in RA patients promotes EPC homing and angiogenesis. EPC migration and tube formation assays examined the effects of CXCL13-controlled homing and angiogenesis [5]. Migratory activity, as well as the formation and reorganization of capillary-like network structures, was significantly greater in EPCs incubated with RA synovial fluid compared with EPCs incubated with OA synovial fluid (Fig. 1D and E). Treatment with CXCL13 antibody dramatically diminished the effects of RA synovial fluid upon EPC migration and tube formation (VEGF-induced EPC migration and tube formation served as the positive control) (Fig. 1D and E). To confirm whether CXCL13 from RA patients increases angiogenesis in vivo, Matrigel containing RA or OA synovial fluid was subcutaneously injected into the flanks of nude male mice. Vessel formation was enhanced by a greater amount by RA synovial fluid compared with OA synovial fluid, according to hemoglobin content of the Matrigel plugs measured using the Drabkin’s method and immunohistochemistry (IHC) staining of vessel marker (CD31 and VEGF) expression (Fig. 1F–H) and the quantitative data of the positive new vessels for VEGF and CD31 immunohistochemistry showed in Fig. 1I. Incubation with CXCL13 antibody downregulated vessel synthesis induced by RA synovial fluid (Fig. 1F–I), indicating that high levels of CXCL13 expression in RA patients induce EPC homing and angiogenesis.

### CXCL13 enhances angiogenesis in vivo

**Fig. 2:**
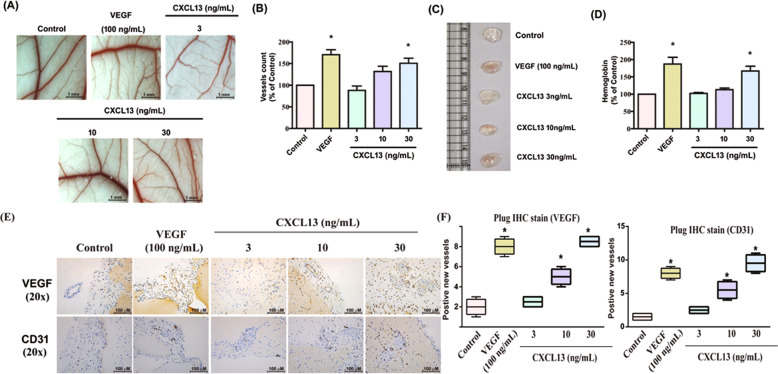
CXCL13 increases angiogenesis in vivo. **A** and **B** Matrigel plugs containing CXCL13 (3–30 ng/ml) or VEGF was applied to 6-day-old fertilized chick embryos for 4 days. CAMs were examined by microscopy and photographed, and vessels were counted (*n* = 6). **C** Matrigel plugs containing CXCL13 or VEGF were subcutaneously injected into the flanks of nude mice (*n* = 6). **D** After 7 days, the plugs were photographed and hemoglobin levels were quantified. **E** Plug specimens were immunostained with antibodies against CD31 and VEGF (*n* = 5). **F** The new positive new vessels of **E** were quantified. *p < 0.05 versus the control group.

To directly examine whether CXCL13 acts as an angiogenic factor in vivo, the chick chorioallantoic membrane (CAM) assay was used. Matrigel was mixed with CXCL13 and placed onto the surface of the CAMs. CXCL13 dose-dependently synthesized new capillaries (VEGF-increased vessel formation served as the positive control) (Fig. 2A and B). The Matrigel plug assay also demonstrated that CXCL13 upregulated microvessel formation, as determined by hemoglobin content, expression of vessel markers (CD31 and VEGF), and the quantitative data of the positive new vessels (Fig. 2C–F). Thus, CXCL13 served as an angiogenic factor and promoted angiogenesis in vivo.

### CXCL13 facilitates VEGF expression and angiogenesis in EPCs via the CXCR5 receptor

**Fig. 3:**
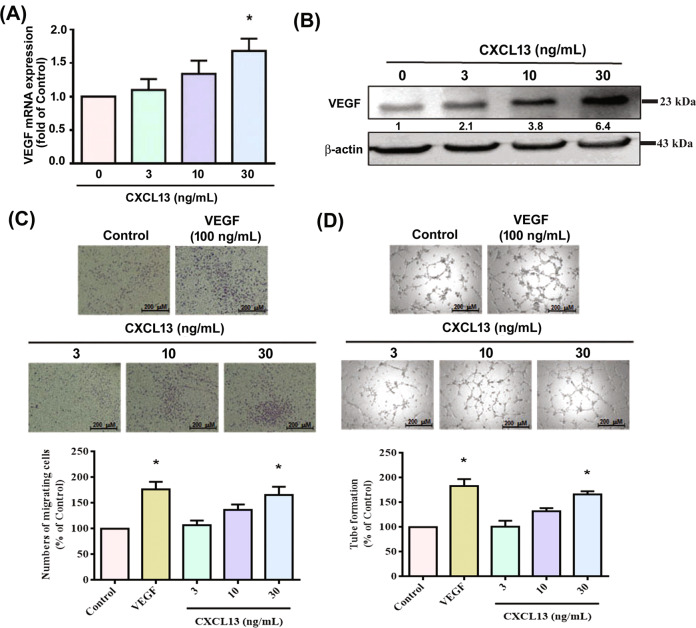
CXCL13 increases VEGF production and EPC migration as well as tube formation. **A** and **B** EPCs were incubated for 24 h with CXCL13 (3–30 ng/ml); VEGF expression was quantified by qPCR (*n* = 6) and Western blot (*n* = 4). (**C**&**D**) EPCs were incubated for 24 h with CXCL13 (3–30 ng/ml); cell migration (*n* = 5) and tube formation (*n* = 5) were measured. *p < 0.05 versus the control group.

**Fig. 4:**
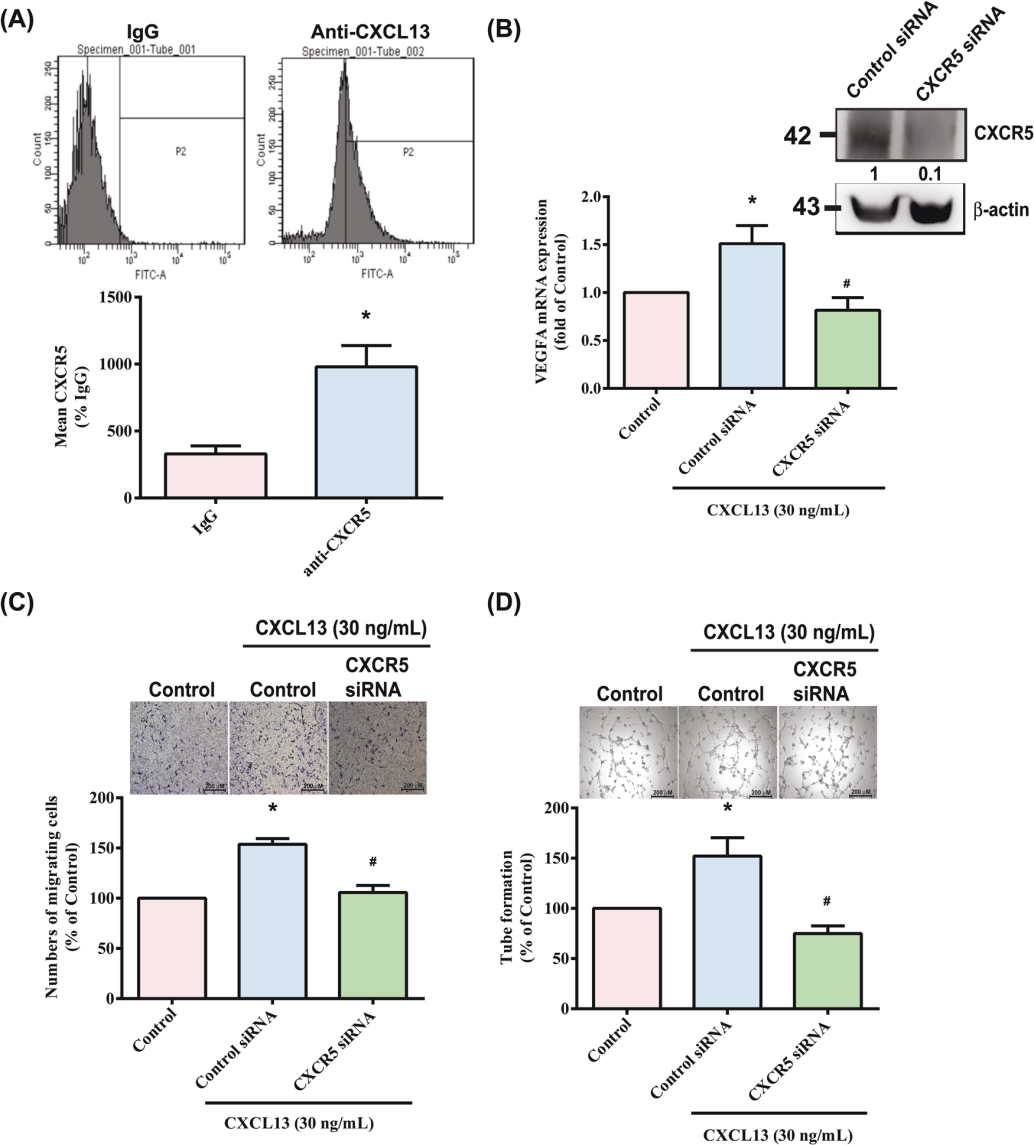
The CXCR5 receptor regulates CXCL13-induced migration and tube formation in EPCs. **A** Cell surface expression of the CXCR5 receptor was examined by flow cytometry (*n* = 4). EPCs were transfected with CXCR5 siRNA, the CXCR5, and β-actin for examination by Western blot (**B**; upper panel) (*n* = 3). EPCs were transfected with CXCR5 siRNA before being stimulated with CXCL13. VEGF expression was examined by qPCR (**B**; lower panel) (*n* = 6), and EPC cell migration (*n* = 5) and tube formation were measured (*n* = 5) (**C**&**D**). *p < 0.05 versus the control group; ^#^*p* < 0.05 versus the CXCL13-treated group.

VEGF is an important angiogenic factor that promotes the growth of new capillaries from pre-existing vessels, regulating angiogenesis during the development of RA disease [2, 5]. We, therefore, examined whether VEGF regulates CXCL13-mediated EPC homing and angiogenesis. CXCL13 dose-dependently increased VEGF mRNA and protein expression in circulating EPCs (Fig. 3A and B) and induced EPC migration and tube formation (Fig. 3C and D). Knockdown of VEGF in EPCs blocked CXCL13 treatment-induced promotion of EPC homing and angiogenesis (Supplementary Fig. S1), indicating that CXCL13 facilitates VEGF-dependent EPC homing and angiogenesis. When we investigated levels of CXCR5 expression in circulating EPCs, flow cytometry data revealed that EPCs expressed the CXCR5 receptor (Fig. 4A). Transfection of EPCs with CXCR5 short interfering RNAi (siRNA) antagonized CXCL13-induced increases in VEGF synthesis and migration, as well as tube formation (Fig. 4B–D). Thus, the CXCR5 receptor mediates CXCL13-promoted VEGF expression and angiogenesis in EPCs.

### The PLC, MEK, and AP-1 signaling cascades regulate CXCL13-promoted VEGF expression and angiogenesis in EPCs

**Fig. 5:**
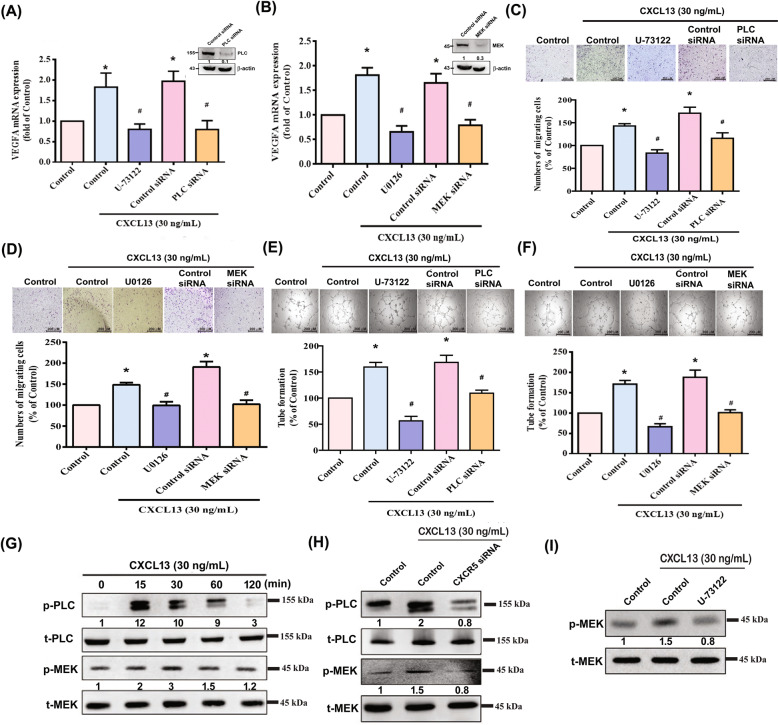
PLC and MEK activation regulate CXCL13-induced VEGF expression and angiogenesis in EPCs. **A**–**F** EPCs were pretreated with PLC (U73122) or MEK (U0126) inhibitors or transfected with PLC and MEK siRNAs then stimulated with CXCL13. VEGF expression (*n* = 6), cell migration (*n* = 5), and tube formation (*n* = 5) were measured. **G** After treating EPCs with CXCL13, PLC and MEK phosphorylation was examined by Western blot (*n* = 4). **H** and **I** EPCs were pretreated with a PLC (U73122) inhibitor, or transfected with CXCR5 siRNA, then stimulated with CXCL13. PLC and MEK phosphorylation was examined by Western blot (*n* = 4). *p < 0.05 versus the control group; ^#^*p* < 0.05 versus the CXCL13-treated group.

The PLC and MEK signaling pathways control various cellular functions, including angiogenesis [17, 18]. We, therefore, sought to determine how these pathways affect CXCL13-induced upregulation of VEGF synthesis and angiogenesis. Treatment of EPCs with PLC (U73122) and MEK (U0126) inhibitors or their siRNAs reduced the effects of CXCL13 upon VEGF expression (Fig. 5A and B) and inhibited CXCL13-induced upregulation of EPC migration and tube formation (Fig. 5C–F). Incubating the EPCs with CXCL13 induced PLC and MEK phosphorylation (Fig. 5G). The CXCR5 siRNA reversed CXCL13-induced promotion of PLC and MEK phosphorylation (Fig. 5H), while U73122 antagonized CXCL13-mediated MEK phosphorylation (Fig. 5I), indicating that CXCL13 promotes VEGF expression and angiogenesis in EPCs via CXCR5, PLC, and MEK signaling.

**Fig. 6:**
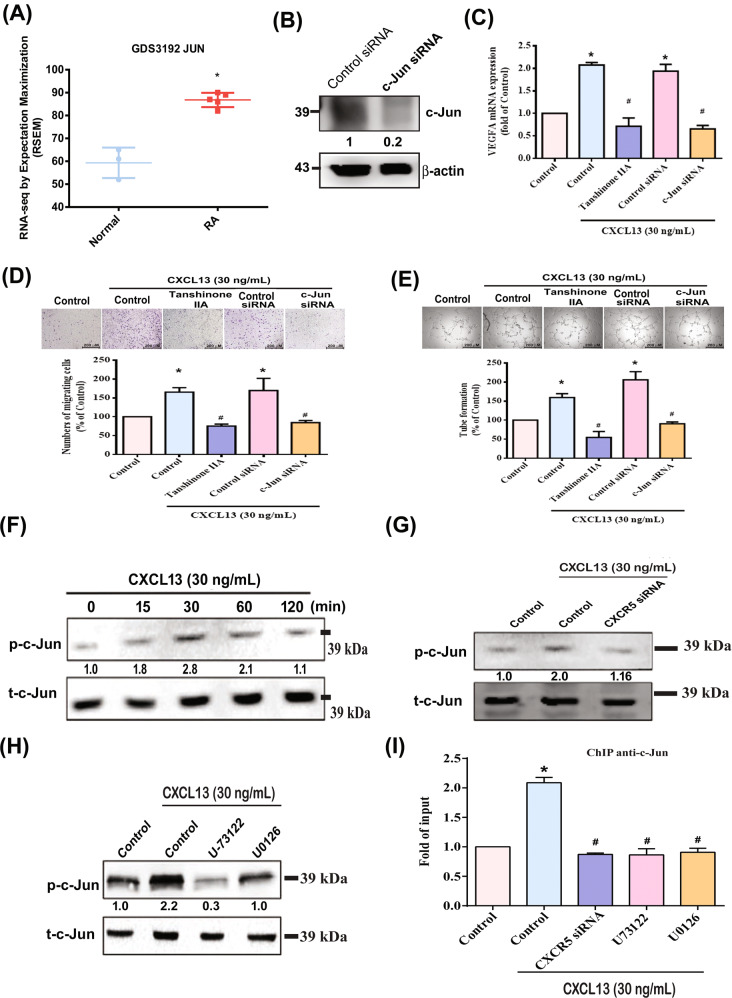
The transcriptional factor AP-1 mediates CXCL13-induced VEGF expression and angiogenesis in EPCs. **A** Expression level of c-Jun in normal healthy individuals, as well as RA patients, were retrieved from the GEO database (accession code: GDS3192). **B** EPCs were transfected with c-Jun siRNA, c-Jun, and β-actin for examination by Western blot (*n* = 4). **C**–**E** EPCs were pretreated with an AP-1 inhibitor (tanshinone IIA), or transfected with c-Jun siRNA, then stimulated with CXCL13. VEGF expression (*n* = 6), cell migration (*n* = 5), and tube formation (*n* = 5) were measured. **F** After treating EPCs with CXCL13, c-Jun phosphorylation was examined by Western blot (*n* = 3). **G** and **H** EPCs were pretreated with PLC (U73122) or MEK (U0126) inhibitors or transfected with CXCR5 siRNA, then stimulated with CXCL13. PLC and MEK phosphorylation was examined by Western blot (*n* = 3). **I** EPCs was transfected with CXCR5 siRNA or pretreated with U73122 or U0126 inhibitors, then stimulated with CXCL13, before undergoing the chromatin immunoprecipitation assay. Chromatin was immunoprecipitated with anti-c-Jun and quantified by qPCR (*n* = 3). *p < 0.05 versus the control group; ^#^*p* < 0.05 versus the CXCL13-treated group.

The transcription factor AP-1 is expressed by different cells and tissues and plays a critical role in mediating the transcription of numerous genes in immune and inflammatory responses [19–22]. The promoter region of human VEGF contains the AP-1-binding site [23, 24]; its activation regulates endothelial cell migration and tube formation by the MAPK or PKC pathways [18, 25, 26]. As shown in Fig. 6A, analysis of records from the GEO database revealed higher c-Jun levels in RA patients than in healthy individuals, and c-Jun expression was identified in EPCs (Fig. 6A and B). Treatment of EPCs with an AP-1 inhibitor (tanshinone IIA) or transfection with a c-Jun siRNA reversed CXCL13-induced upregulation of VEGF expression and angiogenesis (Fig. 6B–E). CXCL13 also facilitated the phosphorylation of c-Jun (Fig. 6F), while CXCR5 siRNA, U73122, and U0126 all abolished CXCL13-increased promotion of c-Jun phosphorylation (Fig. 6G and H).

We further explored whether the PLC and MEK signaling pathway was involved in CXCL13-induced AP-1 transcriptional activation, using the chromatin immunoprecipitation assay to assess in vivo recruitment of AP-1 to the VEGF promoter. As shown in Fig. 6I, the binding of c-Jun to the AP-1 elements by CXCL13 was reduced by CXCR5 siRNA, U73122, and U0126. These results indicate that CXCL13 increases AP-1 activation through CXCR5, PLC, and MEK signaling.

### Inhibition of CXCL13 reduces EPC homing and angiogenesis as well as arthritis severity in vivo

**Fig. 7:**
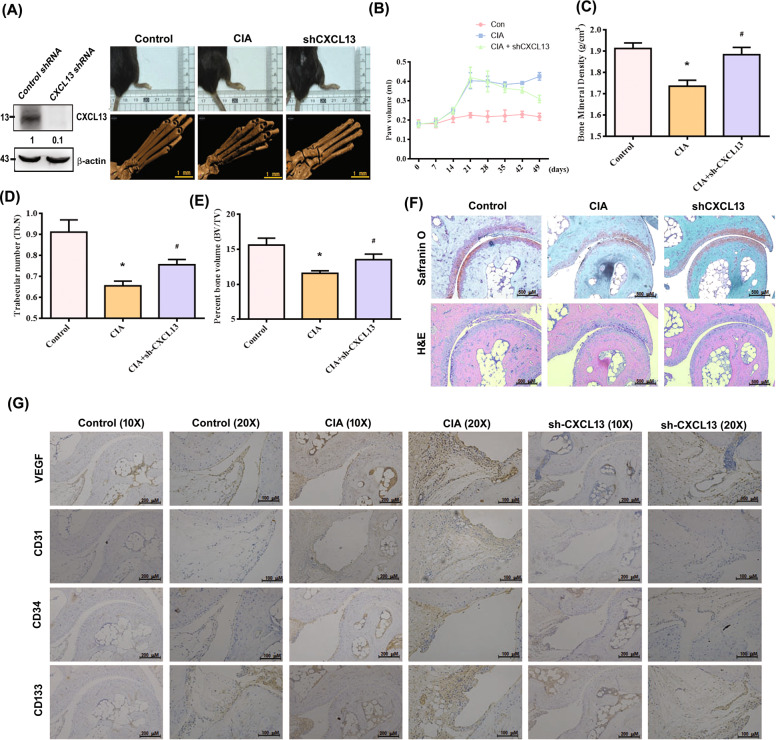
CXCL13 knockdown reduces EPC homing and angiogenesis as well as the severity of RA in vivo. CIA mice received intra-articular injections of 7.1 × 10^6^ PFU CXCL13 shRNA (*n* = 6) or control shRNA (*n* = 6) on day 14 and were euthanized on day 49. **A** Representative micro-CT image of the hind paws was taken on day 49. **B** A digital plethysmometer quantified the amounts of hind paw swelling. **C**–**E** Micro-CT SkyScan Software quantified BMD, trabecular numbers, and bone volume (*n* = 6). **F** and **G** Histological sections of ankle joints were stained with H&E or Safranin O and immunostained with VEGF, CD31, CD34, and CD133 (*n* = 6). *p < 0.05 versus the control group; ^#^*p* < 0.05 versus the CXCL13-treated group.

To explore the therapeutic effect of CXCL13, we used a short hairpin RNA (shRNA) to knock down CXCL13 expression in CIA mice. Compared with controls, CIA mice exhibited significant paw swelling that improved after administration of CXCL13 shRNA (Fig. 7A and B). Micro-computed tomography (micro-CT) images of the hind paws showed that CXCL13 shRNA reversed CIA-induced reductions in bone mineral density (BMD), bone volume (BV), and trabecular (Tb) numbers (Fig. 7A–E). Moreover, CIA mice exhibited lower cartilage thicknesses, as indicated by H&E and Safranin-O/Fast-green staining (Fig. 7F). CXCL13 shRNA reversed CIA-induced cartilage degradation (Fig. 7F). According to IHC staining data, levels of vessel markers (CD31 and VEGF) and EPC markers (CD34 and CD133) were markedly higher in CIA mice than in controls. Notably, CXCL13 shRNA treatment antagonized CIA-induced upregulation of CD31, VEGF, CD34, and CD133 expression (Fig. 7G). These results indicate that inhibiting CXCL13 lowers EPC homing and angiogenesis as well as disease activity in CIA-induced arthritis.

## Discussion

RA is widely recognized for its manifestations of synovial inflammation and joint destruction [1, 27, 28]. The development of RA disease relies upon pannus formation and neovascularization [2], as well as VEGF-induced stimulation of angiogenesis [5]. Previous research has suggested that CXCL13 may serve as a new biomarker of RA, based on higher levels of its expression in RA patients than in healthy controls [12]. In this study, we confirmed higher expression of CXCL13 in synovial fluid from RA patients than from OA patients and healthy individuals, and that CXCL13 attracted EPC homing and angiogenesis via interactions with the CXCR5 receptor. The PLC, MEK, and AP-1 signaling cascades mediate CXCL13-induced upregulations in VEGF expression and angiogenesis in EPCs. Importantly, inhibiting the expression of CXCL13 reduces EPC homing and angiogenesis, reducing the progression of RA in vivo.

EPCs also stimulate new vessel formation [29, 30] and promotion of EPC mobilization by tumor-secreted VEGF facilitates tumor development and angiogenesis [31]. EPC angiogenesis plays a vital role in RA [5, 32]. EPC infiltration into joints has been reported in CIA-induced RA [5]. Here, we observed that compared with OA synovial fluid, RA synovial fluid facilitates EPC infiltration and angiogenesis in vitro and in vivo, indicating that EPC-dependent angiogenesis is an important step during RA progression. Interestingly, the CXCL13 antibody significantly antagonized RA synovial fluid-induced increases in EPC homing and angiogenesis, suggesting that CXCL13 is a vital attractant in EPC-mediated homing and angiogenesis during RA development. Levels of EPC-specific markers were higher in our CIA mouse model than in healthy control mice. CXCL13 shRNA reduced levels of vessel markers and EPC markers and mitigated the severity of RA disease. Thus, inhibition of CXCL13 shows promise as a novel strategy in RA.

Chemokines interact with their specific receptors and thereby regulate migratory, invasive, proliferative, and angiogenic activities during arthritis development [33]. CCL5 binding with the cell surface receptor CCR5 promotes IL-6 production in human OA synovial fibroblasts [34]. Notably, the CXCL12/CXCR4 interaction controls inflammatory cytokine production [35], monocyte infiltration [36], and cartilage degradation [37], all of which play critical roles in the pathogenesis of both OA and RA. In this study, we found that the EPC cell surface expressed the CXCL13-specific receptor CXCR5. CXCL13-induced increases in VEGF production and EPC angiogenesis were markedly inhibited by genetic knockdown of the CXCR5 receptor, indicating that the CXCL13/CXCR5 axis facilitates angiogenic effects of human EPCs.

Activation of the PLC and MEK signaling mechanisms is essential for regulating various cellular functions [38, 39]. The role of PLC in signal transduction is to cleave phosphatidylinositol 4,5-bisphosphonate (PIP_2_) into inositol 1,4,5-trisphosphate (IP3) and diacylglycerol (DAG). PLC signaling is implicated in hematopoiesis and several diseases including diabetes, obesity, and autoimmune disorders [40]. PLC plays important role in the regulation of immune responses; in particular, PLC activity is implicated in ref. [41], while PLC signaling reportedly controls different cellular functions, including angiogenesis [17]. Moreover, the activation of the MAPK signaling pathway is essential in many types of cells for mediating multiple cellular functions [42]. As part of the MAPK cascade, MEK is implicated in the regulation of RA angiogenesis [43, 44]. Our investigations found that PLC and MEK inhibitors reduced CXCL13-enhanced VEGF expression as well as EPC migration and tube formation. This was confirmed by findings from genetic siRNA experiments demonstrating that PLC and MEK mediate the angiogenic effects of CXCL13. Treatment of EPCs with CXCL13 augmented PLC and MEK phosphorylation, while PLC inhibitor treatment inhibited CXCL13-promoted MEK phosphorylation. This suggests that MEK is a downstream molecule of PLC. Likewise, CXCR5 siRNA decreased PLC and MEK phosphorylation after CXCL13 stimulation, suggesting that CXCR5, PLC and MEK activation controls CXCL13-induced increases in VEGF expression and angiogenic activity in human circulating EPCs.

**Fig. 8:**
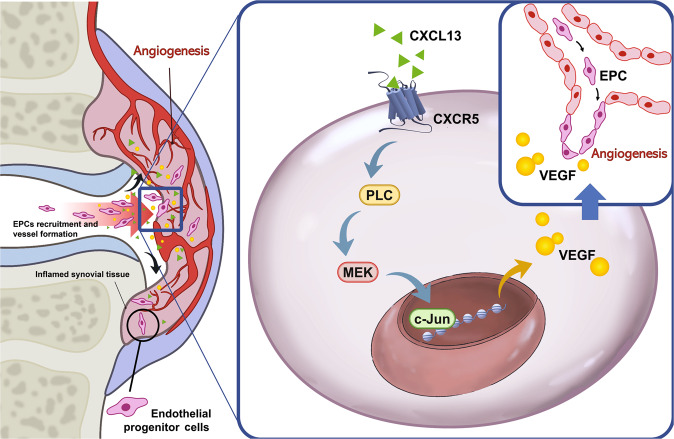
Schematic diagram summarizes the mechanisms in the CXCL13/CXCR5 axis that promote EPC homing and angiogenesis during RA pathogenesis. Accumulation of CXCL13 in the synovium of RA patients promotes VEGF expression, EPC homing, and angiogenesis through the CXCR5 receptor, PLC, MEK, and AP-1 signaling pathways.

AP-1 is an important transcription factor that regulates VEGF transcriptional activity and angiogenesis during the progression of RA [45]. Several lines of evidence have indicated that the activation of AP-1 regulates endothelial cell migration and tube formation by the MAPK or PKC pathways [18, 25, 26]. However, how CXCL13 regulates RA angiogenesis through the PLC/MEK/AP-1 pathway remains unknown. The results of this study show that an AP-1 inhibitor (tanshinone IIA) reduced CXCL13-induced promotion of VEGF synthesis and angiogenesis in human EPCs, which suggests the importance of AP-1 activation in these processes. Stimulation of EPCs with CXCL13 also facilitated c-Jun phosphorylation. The upstream signaling pathways mediating the CXCR5 siRNA, PLC, and MEK inhibitors antagonized CXCL13-induced phosphorylation of c-Jun. These results document how CXCL13 promotes AP-1-regulated VEGF production and angiogenesis in human EPCs via CXCR5, PLC, and MEK signaling mechanisms. We have determined that higher accumulation of CXCL13 in the RA synovium attracts EPC homing and angiogenesis through the PLC, MEK, and AP-1 signaling pathways via binding to the CXCR5 receptor on the EPC surface. Inhibiting CXCL13 antagonized EPC infiltration and angiogenesis, inhibiting RA progression (Fig. 8). The evidence supports the targeting of CXCL13 in RA treatment regimens.

## Materials and methods

### Materials

PLC (SC-58407), MEK (SC-6250), c-Jun (SC-74543), and VEGF (SC-7269) antibodies were purchased from Santa Cruz Biotechnology (CA, USA). p-PLC (2821S), p-MEK (2338S), and p-c-Jun (3270S) antibodies were purchased from Cell Signaling Technology (Danvers, MA, USA). All siRNAs (ON-TARGET*plus*) were obtained from Dharmacon Research (Lafayette, CO, USA). Taqman® one-step PCR Master Mix, qPCR primers, and probes were obtained from Applied Biosystems (Foster City, CA, USA). Beta-actin antibodies and pharmacological inhibitors were obtained from Sigma-Aldrich (St. Louis, MO, USA).

### Human synovial fluid samples

Study approval was granted by the Institutional Review Board of China Medical University Hospital (Taichung, Taiwan) and all patients provided written informed consent before participating in the study. Synovial fluid samples were obtained from patients undergoing total knee arthroplasty for OA or RA.

### Analysis of the GEO database

CXCL13 mRNA levels for normal healthy (*n* = 10) individuals, RA (*n* = 10), and OA (*n* = 10) patients were retrieved from the GEO database (accession code: GDS5401). C-Jun mRNA levels for normally healthy individuals (*n* = 3) and RA patients (*n* = 5) were retrieved from the GEO database (accession code: GDS3192) [46].

### Cell culture

Human EPCs were prepared according to our previous protocols [5, 47, 48], after we had obtained approval from the Institutional Review Board (IRB) of Mackay Medical College, New Taipei City, Taiwan (reference number: P1000002). Peripheral blood was collected from healthy donors after they completed written informed consent forms. Mononuclear cells were isolated from blood components using centrifugation on Ficoll-Paque PLUS (Amersham Biosciences, Uppsala, Sweden). EPCs were characterized and maintained using methods described in our previous reports [32, 49, 50].

### Western blot analysis

Cell lysates were resolved by SDS–PAGE and transferred to Immobilon® PVDF membranes. The blots were blocked with 4% bovine serum albumin (BSA) for 1 h at room temperature and then probed with rabbit anti-human antibodies against primary antibody (1:1000) for 1 h at room temperature. After three washes, the blots were subsequently incubated with donkey anti-rabbit peroxidase-conjugated secondary antibody (1:3000) for 1 h at room temperature. Enhanced chemiluminescent imaging of the blots was visualized using the UVP Biospectrum system (UVP, Upland, CA, USA) [51].

### EPC tube formation

Matrigel (BD Biosciences, Bedford, MA, USA) coated on 48-well plates and EPCs (2 × 10^4^ per 100 μL) were resuspended in MV2 serum-free medium with the indicated CXCL13 concentration and then added to the wells. After 6 h of incubation at 37 °C, EPC tube formation was assessed with a photomicroscope, and each well was photographed at ×200 magnification. The number of tube branches was calculated using MacBiophotonics Image J software (v1.51, National Institutes of Health, USA) [6, 52].

### EPC migration

EPC migration assays were used with Transwell inserts (Corning/Costar, Corning, NY, USA) in 24-well plates, and 2 × 10^4^ EPCs were applied to the upper chamber with 10% FBS MV2 medium and the culture medium was combined with 20% FBS MV2 complete medium in the lower chamber. After incubating the plates for 16 h at 37 °C in 5% CO_2_, the cells were fixed in 3.7% formaldehyde solution for 15 min and stained with 0.05% crystal violet in phosphate-buffered saline (PBS) for 15 min. Cells on the upper side of the filters were removed with cotton-tipped swabs, and the filters were washed with PBS. Cells on the underside of the filters were examined and counted under a microscope [6, 17].

### The chick chorioallantoic membrane (CAM) assay

The CAM assay was adapted from a previously described assay [6, 53, 54]. On day 7, CM was collected from CXCL13 treatment EPCs (2 × 10^4^ cells) were deposited in the center of the CAM. At 11 days, CAMs were collected for microscopy and photographic documentation. Angiogenesis was quantified by counting the number of blood vessel branches; at least 10 viable embryos were tested for each treatment. All animal work was done in accordance with a protocol approved by the China Medical University (Taichung, Taiwan) Institutional Animal Care and Use Committee.

### In vivo matrigel plug assay

The Matrigel plug angiogenesis assay was adapted from a previously described assay [6, 53, 54]. Four-week-old nude male mice received a single subcutaneous injection of Matrigel (300 μL) containing RA or OA synovial fluid. After 7 days, the Matrigel plugs were harvested and hemoglobin concentrations were measured. The plugs were embedded in paraffin and processed for IHC staining for vessel markers VEGF and CD31.

### CIA mouse model

C57BL/6J, 6 weeks, male mice were purchased from the National Laboratory Animal Centre (Taipei, Taiwan) and CIA mouse model protocol followed that detailed in previously published work. An emulsion containing bovine type II collagen (CII, Chondrex, Redmond, WA, USA) and IFA Freund’s incomplete adjuvant (Sigma-Aldrich, St. Louis, MO, USA) was intradermally injected into each mouse tail root on day 0; we then intra-articularly injected the same amount on day 14 according to the improved method [6, 54, 55].

Arthritis in CIA mice develops reliably within 6 weeks. Following both immunizations, the mice have randomly separated three groups (Sham, CIA, and CIA + sh CXCL13) given weekly intra-articular injections of ~7.1 × 10^6^ plaque-forming units (PFU) of control (*n* = 6) or CXCL13 shRNA (*n* = 6). Upon sacrifice after 49 days of treatment, phalanges and ankle joints were removed from each mouse then fixed in 4% paraformaldehyde for micro-CT analysis.

### Statistical analysis

All statistical analyses were carried out using GraphPad Prism 5.0 (GraphPad Software) and all values are expressed as the mean ± SD. Differences between selected pairs from the experimental groups were analyzed for statistical significance using the paired sample *t*-test for in vitro analyses and by one-way ANOVA followed by Bonferroni testing for in vivo analyses.

## Supplementary information

Supplementary data

Supplementary figure S1

Supplementary figure S2

Supplementary figure S3

## Data Availability

The data generated and analyzed will be made from the corresponding author on reasonable request. Full, uncropped Western blot images are now provided in the Supplementary files (Figs. S2 and S3).

## References

[1] Smolen JS, Aletaha D, McInnes IB (2016). Rheumatoid arthritis. Lancet.

[2] MacDonald IJ, Liu SC, Su CM, Wang YH, Tsai CH, Tang CH (2018). Implications of angiogenesis involvement in arthritis. Int J Mol Sci.

[3] Asahara T, Masuda H, Takahashi T, Kalka C, Pastore C, Silver M (1999). Bone marrow origin of endothelial progenitor cells responsible for postnatal vasculogenesis in physiological and pathological neovascularization. Circ Res.

[4] Peplow PV (2014). Influence of growth factors and cytokines on angiogenic function of endothelial progenitor cells: a review of in vitro human studies. Growth Factors.

[5] Su CM, Hsu CJ, Tsai CH, Huang CY, Wang SW, Tang CH (2015). Resistin promotes angiogenesis in endothelial progenitor cells through inhibition of microRNA206: potential implications for rheumatoid arthritis. Stem Cells.

[6] Chen CY, Su CM, Hsu CJ, Huang CC, Wang SW, Liu SC (2017). CCN1 promotes VEGF production in osteoblasts and induces endothelial progenitor cell angiogenesis by inhibiting miR-126 expression in rheumatoid arthritis. J Bone Miner Res.

[7] Finch DK, Ettinger R, Karnell JL, Herbst R, Sleeman MA (2013). Effects of CXCL13 inhibition on lymphoid follicles in models of autoimmune disease. Eur J Clin Investig.

[8] Hussain M, Adah D, Tariq M, Lu Y, Zhang J, Liu J (2019). CXCL13/CXCR5 signaling axis in cancer. Life Sci.

[9] Armas-González E, Domínguez-Luis MJ, Díaz-Martín A, Arce-Franco M, Castro-Hernández J, Danelon G (2018). Role of CXCL13 and CCL20 in the recruitment of B cells to inflammatory foci in chronic arthritis. Arthritis Res Ther.

[10] Humby F, Bombardieri M, Manzo A, Kelly S, Blades MC, Kirkham B (2009). Ectopic lymphoid structures support ongoing production of class-switched autoantibodies in rheumatoid synovium. PLoS Med.

[11] Yoshitomi H (2020). CXCL13-producing PD-1(hi)CXCR5(-) helper T cells in chronic inflammation. Immunol Med.

[12] Meeuwisse CM, van der Linden MP, Rullmann TA, Allaart CF, Nelissen R, Huizinga TW (2011). Identification of CXCL13 as a marker for rheumatoid arthritis outcome using an in silico model of the rheumatic joint. Arthritis Rheum.

[13] Mandik-Nayak L, Huang G, Sheehan KC, Erikson J, Chaplin DD (2001). Signaling through TNF receptor p55 in TNF-alpha-deficient mice alters the CXCL13/CCL19/CCL21 ratio in the spleen and induces maturation and migration of anergic B cells into the B cell follicle. J Immunol.

[14] Wengner AM, Höpken UE, Petrow PK, Hartmann S, Schurigt U, Bräuer R (2007). CXCR5- and CCR7-dependent lymphoid neogenesis in a murine model of chronic antigen-induced arthritis. Arthritis Rheum.

[15] Klimatcheva E, Pandina T, Reilly C, Torno S, Bussler H, Scrivens M (2015). CXCL13 antibody for the treatment of autoimmune disorders. BMC Immunol.

[16] Bechman K, Dalrymple A, Southey-Bassols C, Cope AP, Galloway JB (2020). A systematic review of CXCL13 as a biomarker of disease and treatment response in rheumatoid arthritis. BMC Rheumatol.

[17] Wang LH, Tsai HC, Cheng YC, Lin CY, Huang YL, Tsai CH (2017). CTGF promotes osteosarcoma angiogenesis by regulating miR-543/angiopoietin 2 signaling. Cancer Lett.

[18] Wang CQ, Lin CY, Huang YL, Wang SW, Wang Y, Huang BF (2019). Sphingosine-1-phosphate promotes PDGF-dependent endothelial progenitor cell angiogenesis in human chondrosarcoma cells. Aging.

[19] Xu Y, Nowrangi D, Liang H, Wang T, Yu L, Lu T (2020). DKK3 attenuates JNK and AP-1 induced inflammation via Kremen-1 and DVL-1 in mice following intracerebral hemorrhage. J Neuroinflamm.

[20] Uluckan O, Guinea-Viniegra J, Jimenez M, Wagner EF (2015). Signalling in inflammatory skin disease by AP-1 (Fos/Jun). Clin Exp Rheumatol.

[21] Shiozawa S, Tsumiyama K (2009). Pathogenesis of rheumatoid arthritis and c-Fos/AP-1. Cell Cycle.

[22] Yanagida K, Engelbrecht E, Niaudet C, Jung B, Gaengel K, Holton K (2020). Sphingosine 1-phosphate receptor signaling establishes AP-1 gradients to allow for retinal endothelial cell specialization. Dev Cell.

[23] Qin L, Xu Y, Xu Y, Ma G, Liao L, Wu Y (2015). NCOA1 promotes angiogenesis in breast tumors by simultaneously enhancing both HIF1alpha- and AP-1-mediated VEGFa transcription. Oncotarget.

[24] Lee HP, Lin CY, Shih JS, Fong YC, Wang SW, Li TM (2015). Adiponectin promotes VEGF-A-dependent angiogenesis in human chondrosarcoma through PI3K, Akt, mTOR, and HIF-alpha pathway. Oncotarget.

[25] Jia J, Ye T, Cui P, Hua Q, Zeng H, Zhao D (2016). AP-1 transcription factor mediates VEGF-induced endothelial cell migration and proliferation. Microvasc Res.

[26] Jones MK, Tsugawa K, Tarnawski AS, Baatar D (2004). Dual actions of nitric oxide on angiogenesis: possible roles of PKC, ERK, and AP-1. Biochem Biophys Res Commun.

[27] McInnes IB, Schett G (2011). The pathogenesis of rheumatoid arthritis. N Engl J Med.

[28] Catrina AI, Svensson CI, Malmstrom V, Schett G, Klareskog L (2017). Mechanisms leading from systemic autoimmunity to joint-specific disease in rheumatoid arthritis. Nat Rev Rheumatol.

[29] Patel J, Donovan P, Khosrotehrani K (2016). Concise review: functional definition of endothelial progenitor cells: a molecular perspective. Stem Cells Transl Med.

[30] Kiewisz J, Kaczmarek MM, Pawlowska A, Kmiec Z, Stompor T (2016). Endothelial progenitor cells participation in cardiovascular and kidney diseases: a systematic review. Acta Biochim Pol.

[31] Peters BA, Diaz LA, Polyak K, Meszler L, Romans K, Guinan EC (2005). Contribution of bone marrow-derived endothelial cells to human tumor vasculature. Nat Med.

[32] Chien SY, Huang CY, Tsai CH, Wang SW, Lin YM, Tang CH (2016). Interleukin-1beta induces fibroblast growth factor 2 expression and subsequently promotes endothelial progenitor cell angiogenesis in chondrocytes. Clin Sci.

[33] Szekanecz Z, Szucs G, Szanto S, Koch AE (2006). Chemokines in rheumatic diseases. Curr Drug Targets.

[34] Tang CH, Hsu CJ, Fong YC (2010). The CCL5/CCR5 axis promotes interleukin-6 production in human synovial fibroblasts. Arthritis Rheum.

[35] Chen HT, Tsou HK, Hsu CJ, Tsai CH, Kao CH, Fong YC (2011). Stromal cell-derived factor-1/CXCR4 promotes IL-6 production in human synovial fibroblasts. J Cell Biochem.

[36] Cecchinato V, D'Agostino G, Raeli L, Nerviani A, Schiraldi M, Danelon G (2018). Redox-mediated mechanisms fuel monocyte responses to CXCL12/HMGB1 in active rheumatoid arthritis. Front Immunol.

[37] Chiu YC, Yang RS, Hsieh KH, Fong YC, Way TD, Lee TS (2007). Stromal cell-derived factor-1 induces matrix metalloprotease-13 expression in human chondrocytes. Mol Pharmacol.

[38] Katan M, Cockcroft S (2020). Phospholipase C families: common themes and versatility in physiology and pathology. Prog Lipid Res.

[39] Kun E, Tsang YTM, Ng CW, Gershenson DM, Wong KK (2020). MEK inhibitor resistance mechanisms and recent developments in combination trials. Cancer Treat Rev.

[40] Bae YS, Lee HY, Jung YS, Lee M, Suh PG (2017). Phospholipase Cgamma in Toll-like receptor-mediated inflammation and innate immunity. Adv Biol Regul.

[41] Li H, Hao Z, Zhao L, Liu W, Han Y, Bai Y (2016). Comparison of molecular mechanisms of rheumatoid arthritis and osteoarthritis using gene microarrays. Mol Med Rep.

[42] Wee P, Wang Z (2017). Epidermal growth factor receptor cell proliferation signaling pathways. Cancers.

[43] Hu SL, Huang CC, Tzeng TT, Liu SC, Tsai CH, Fong YC (2020). S1P promotes IL-6 expression in osteoblasts through the PI3K, MEK/ERK and NF-kappaB signaling pathways. Int J Med Sci.

[44] Elshabrawy HA, Chen Z, Volin MV, Ravella S, Virupannavar S, Shahrara S (2015). The pathogenic role of angiogenesis in rheumatoid arthritis. Angiogenesis.

[45] Le Rossignol S, Ketheesan N, Haleagrahara N (2018). Redox-sensitive transcription factors play a significant role in the development of rheumatoid arthritis. Int Rev Immunol.

[46] Lee H-P, Wu YC, Chen BC, Liu SC, Li TM, Huang WC (2020). Soya-cerebroside reduces interleukin production in human rheumatoid arthritis synovial fibroblasts by inhibiting the ERK, NF-κB and AP-1 signalling pathways. Food Agr Immunol.

[47] Wu MH, Huang CY, Lin JA, Wang SW, Peng CY, Cheng HC (2014). Endothelin-1 promotes vascular endothelial growth factor-dependent angiogenesis in human chondrosarcoma cells. Oncogene.

[48] Lee H-P, Wang SW, Wu YC, Tsai CH, Tsai FJ, Chung JG (2019). Glucocerebroside reduces endothelial progenitor cell-induced angiogenesis. Food Agric Immunol.

[49] Lee H-P, Wang SW, Wu YC, Lin LW, Tsai FJ, Yang JS (2020). Soya-cerebroside inhibits VEGF-facilitated angiogenesis in endothelial progenitor cells. Food Agric Immunol.

[50] Lee H-P, Chen PC, Wang SW, Fong YC, Tsai CH, Tsai FJ (2019). Plumbagin suppresses endothelial progenitor cell-related angiogenesis in vitro and in vivo. J Funct Foods.

[51] Liu JF, Lee CW, Tsai MH, Tang CH, Chen PC, Lin LW (2018). Thrombospondin 2 promotes tumor metastasis by inducing matrix metalloproteinase-13 production in lung cancer cells. Biochem Pharmacol.

[52] Su CM, Wang IC, Liu SC, Sun Y, Jin L, Wang SW (2017). Hypoxia induced mitogenic factor (HIMF) triggers angiogenesis by increasing interleukin-18 production in myoblasts. Sci Rep.

[53] Wang CQ, Huang YW, Wang SW, Huang YL, Tsai CH, Zhao YM (2017). Amphiregulin enhances VEGF-A production in human chondrosarcoma cells and promotes angiogenesis by inhibiting miR-206 via FAK/c-Src/PKCdelta pathway. Cancer Lett.

[54] Su CM, Chiang YC, Huang CY, Hsu CJ, Fong YC, Tang CH (2015). Osteopontin promotes oncostatin M production in human osteoblasts: implication of rheumatoid arthritis therapy. J Immunol.

[55] Hu SL, Chang AC, Huang CC, Tsai CH, Lin CC, Tang CH (2017). Myostatin promotes interleukin-1beta expression in rheumatoid arthritis synovial fibroblasts through inhibition of miR-21-5p. Front Immunol.

