# Supplementary material for: CXCL13/CXCR5 axis facilitates endothelial progenitor cell homing and angiogenesis during rheumatoid arthritis progression
Source: Cell Death Dis. 2021 Sep 13;12(9):846. doi: 10.1038/s41419-021-04136-2 (PMC8437941; doi:10.1038/s41419-021-04136-2)
Supplement: Supplementary file 1 — Supplementary data [file 41419_2021_4136_MOESM1_ESM.doc]

**Supplemental methods**

**Quantitative real-time PCR (qPCR) analysis of mRNA**

Total RNA was extracted from EPCs using TRIzol reagent and then reverse-transcribed into cDNA using oligo(dT) primers. Real-time quantitative PCR (qPCR) analysis was performed using One-Step RT-PCR Master Mix (Applied Biosystems, CA, USA), according to an established protocol (1-3). Sequence-specific primers were used to add 2 µL of cDNA template to each 25-μL reaction. Glyceraldehyde 3-phosphate dehydrogenase (GAPDH) was used as an endogenous control to normalize expression data (Applied Biosystems). qPCR assays were carried out in triplicate in a StepOnePlus sequence detection system (Applied Biosystems, CA, USA). The cycling conditions were as follows: initial 10-min polymerase activation at 95°C followed by 40 cycles at 95°C for 15 s and 60°C for 60 s. The threshold was set above the non-template control background and within the linear phase of the target gene amplification to calculate the cycle number at which the transcript was detected (denoted as CT). The primer sequences used were defined as VEGF-A forward primer: (GCAGAATCATCACGAAGTGG); reverse primer: (GCATGGTGATGTTGGACTCC); GAPDH forward primer: (ACCACAGTCCATGCCATCAC); and reverse primer: (TCCACCACCCTGTTGCTGTA).

**ELISA assay**

EPCs were cultured in 24-well plates until they reached 90% confluence before being changed to serum-free medium, in which they were treated with CXCL13 for 24 h with or without the transfection of siRNAs or inhibitors. The conditioned medium (CM) was collected and VEGF levels were quantified with the VEGF ELISA kit (Peprotech, Rocky Hill, NJ, USA), following the manufacturer’s protocol.

Synovial fluid was collected from patients with OA or RA. VEGF levels were quantified using the VEGF ELISA kit.

**Chromatin immunoprecipitation assay**

Chromatin immunoprecipitation analysis was performed as described previously (4). The primers 5’-AAGCTGTGAGCCTGGAGAAG-3’ and 5’-CACTGTGGAGTCTGGCAAAA-3’ were utilized to amplify qPCR across the human VEGF promoter region contain AP-1 binding site (5).

**Supplemental Figure legends**

**Supplemental Figure S1. VEGF is essential in CXCL13-induced promotion of EPC homing and angiogenesis.** (A) EPCs were transfected with VEGF siRNA, the VEGF and β-actin expression was examined by Western blot. (B&C) EPCs were transfected with VEGF siRNA before being stimulated with CXCL13. EPC migration and tube formation were measured. Results are expressed as the mean ± S.D. * *p* < 0.05 compared with control.

**Supplemental Figure S2. Uncropped blots for Western blot.** The Fig 3B, 4B, 5A, 5B, 5C, 5H, and 5I uncropped blots.

**Supplemental Figure S3. Uncropped blots for Western blot.** The Fig 6B, 6F, 6G, 6I, 7A, and S1A uncropped blots.

Reference

1. Huang CC, et al. Melatonin attenuates TNF-alpha and IL-1beta expression in synovial fibroblasts and diminishes cartilage degradation: Implications for the treatment of rheumatoid arthritis. Journal of pineal research. 2019;66(3):e12560.

2. Liu SC, et al. Soya-cerebroside reduces IL-1 beta-induced MMP-1 production in chondrocytes and inhibits cartilage degradation: implications for the treatment of osteoarthritis. Food Agr Immunol. 2019;30(1):620-32.

3. Wang M, et al. Thrombospondin enhances RANKL-dependent osteoclastogenesis and facilitates lung cancer bone metastasis. Biochemical pharmacology. 2019;166:23-32.

4. Chiu YC, et al. Peptidoglycan enhances IL-6 production in human synovial fibroblasts via TLR2 receptor, focal adhesion kinase, Akt, and AP-1- dependent pathway. J Immunol. 2009;183(4):2785-92.

5. Qin L, et al. NCOA1 promotes angiogenesis in breast tumors by simultaneously enhancing both HIF1alpha- and AP-1-mediated VEGFa transcription. Oncotarget. 2015;6(27):23890-904.
