# Supplementary figures and images for: CXCL13/CXCR5 axis facilitates endothelial progenitor cell homing and angiogenesis during rheumatoid arthritis progression
Source: Cell Death Dis. 2021 Sep 13;12(9):846. doi: 10.1038/s41419-021-04136-2 (PMC8437941; doi:10.1038/s41419-021-04136-2)

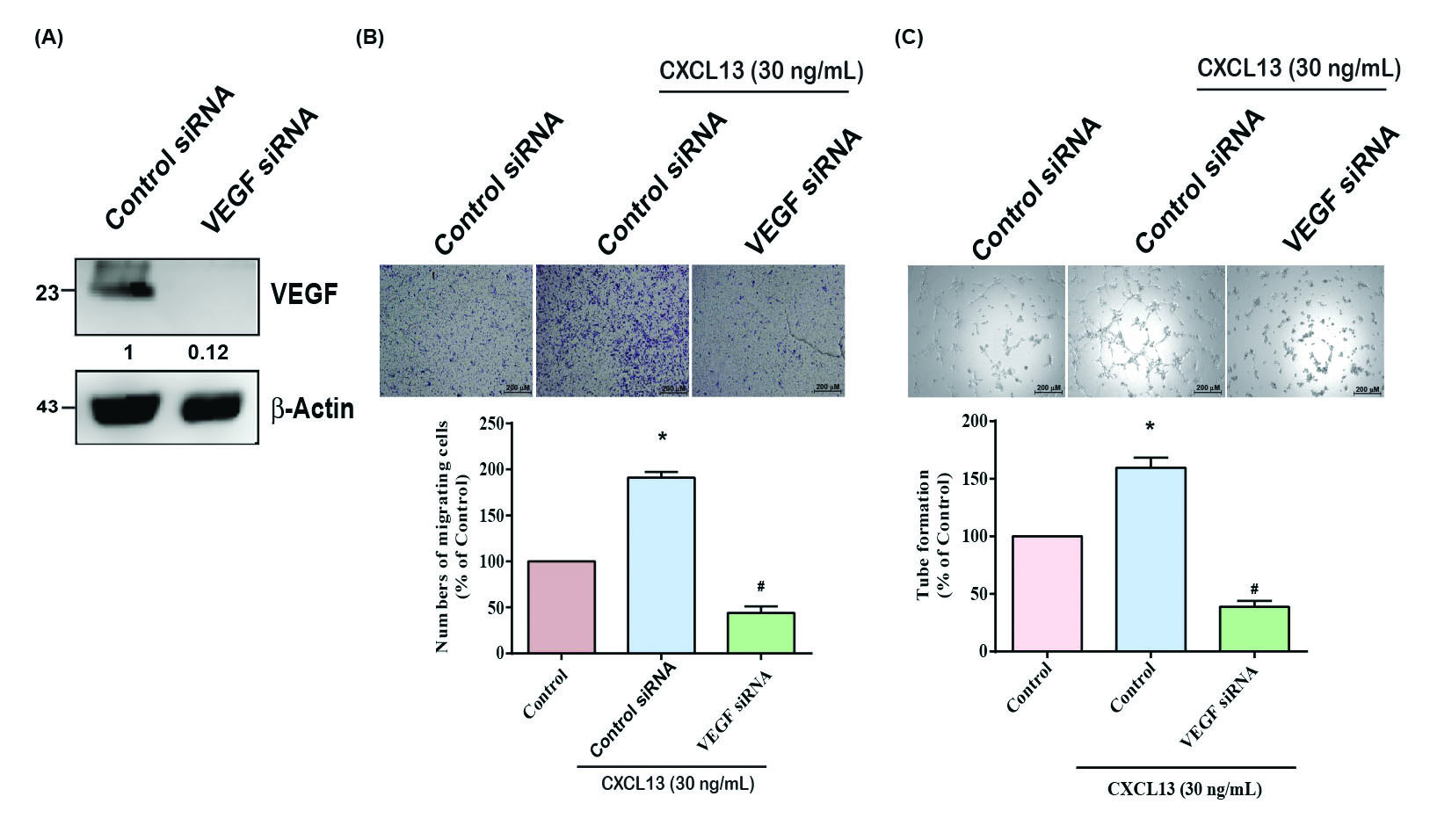

Supplement: Supplementary file 2 — Supplementary figure S1 [file 41419_2021_4136_MOESM2_ESM.tif]

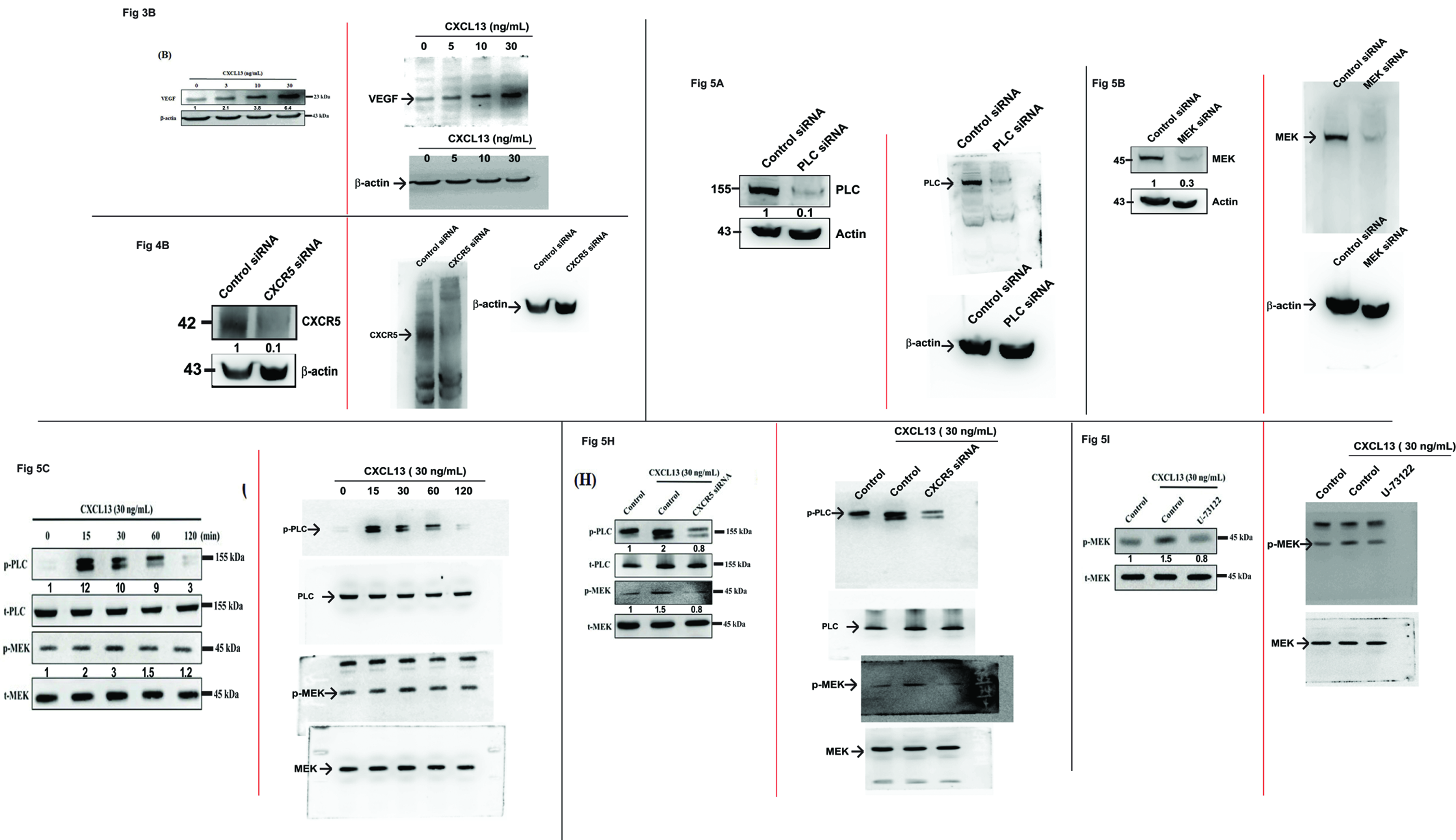

Supplement: Supplementary file 3 — Supplementary figure S2 [file 41419_2021_4136_MOESM3_ESM.tif]

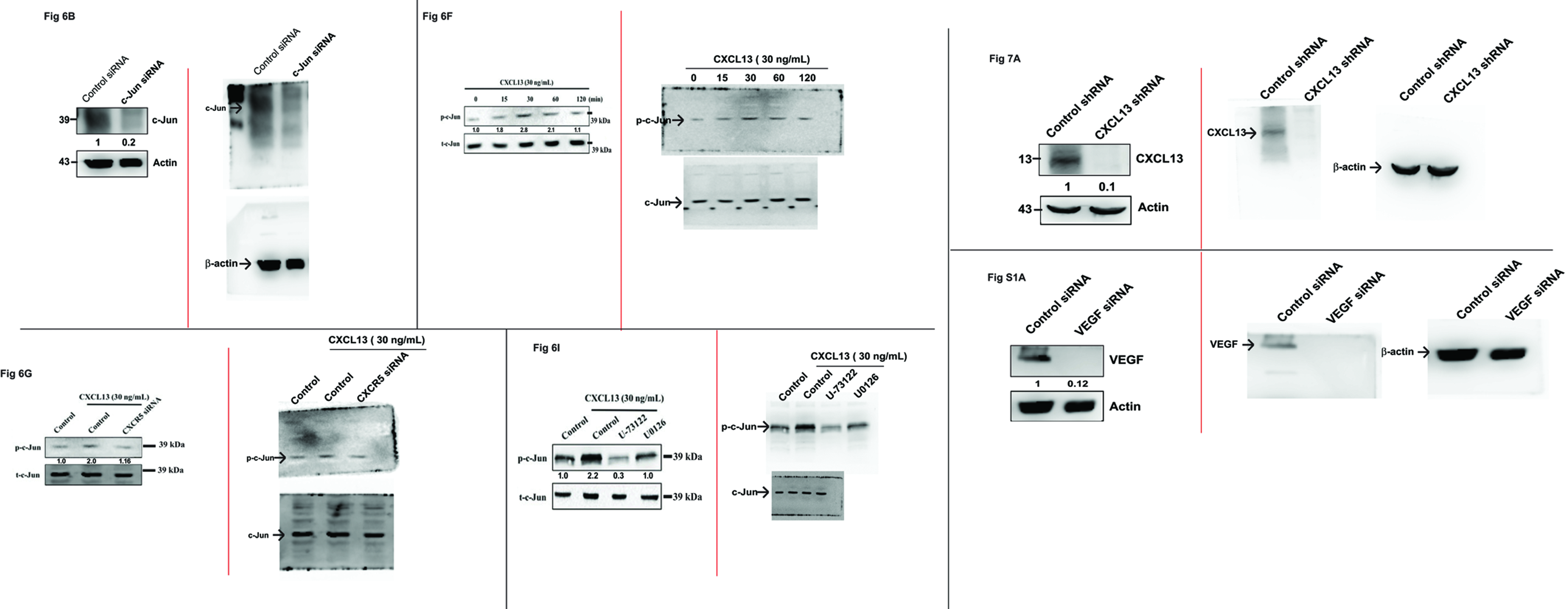

Supplement: Supplementary file 4 — Supplementary figure S3 [file 41419_2021_4136_MOESM4_ESM.tif]
